# The developments and prospects of plant super-pangenomes: Demands, approaches, and applications

**DOI:** 10.1016/j.xplc.2024.101230

**Published:** 2024-12-24

**Authors:** Wenchuang He, XiaoXia Li, Qian Qian, Lianguang Shang

**Affiliations:** 1 Shenzhen Branch, Guangdong Laboratory of Lingnan Modern Agriculture, Genome Analysis Laboratory of the Ministry of Agriculture and Rural Affairs, Agricultural Genomics Institute at Shenzhen, Chinese Academy of Agricultural Sciences, Shenzhen 518120, China; 2 Yazhouwan National Laboratory, Sanya 572024, China; 3 State Key Laboratory of Rice Biology, China National Rice Research Institute, Hangzhou 310006, China; 4 Academician Workstation, National Nanfan Research Institute (Sanya), Chinese Academy of Agricultural Sciences, Sanya 572024, China

**Keywords:** pangenomics, super-pangenome, plant, methodology, molecular breeding

## Abstract

By integrating genomes from different accessions, pangenomes provide a more comprehensive and reference-bias-free representation of genetic information within a population compared to a single reference genome. With the rapid accumulation of genomic sequencing data and the expanding scope of plant research, plant pangenomics has gradually evolved from single-species to multi-species studies. This shift has given rise to the concept of a super-pangenome that covers all genomic sequences within a genus-level taxonomic group. By incorporating both cultivated and wild species, the super-pangenome has greatly enhanced the resolution of research in various areas such as plant genetic diversity, evolution, domestication, and molecular breeding. In this review, we present a comprehensive overview of the plant super-pangenome, emphasizing its development requirements, construction strategies, potential applications, and notable achievements. We also highlight the distinctive advantages and promising prospects of super-pangenomes while addressing current challenges and future directions.

## Teaser

By incorporating a broader range of cultivated and wild species, the plant super-pangenome has substantially advanced multiple research areas. This review systematically summarizes the developments and prospects of plant super-pangenomes, emphasizing their unique value, developmental demands, construction approaches, potential applications, and notable achievements. It further highlights the distinctive advantages, promising outlook, and future directions of this emerging field.

## Introduction

Hailed as the "moon landing project" of life sciences, the Human Genome Project was launched in 1990 with the goal of completely sequencing and analyzing the human genome. Following this, the release of the first reference genomes for the model plants *Arabidopsis* (Arabidopsis Genome Initiative, 2000) and rice (International Rice Genome Sequencing Project, 2005) ushered in the era of genomics in plant research. After decades of development, plant genome assembly has progressed from the chromosome level to telomere-to-telomere (T2T) assemblies (Naish et al., 2021; Song et al., 2021). Most recently, researchers have achieved complete genome-level T2T assemblies for all chromosomes (Chen et al., 2023a; Shang et al., 2023). This progress has resulted in an abundance of genome assemblies across hundreds of species (Sun et al., 2022), substantially contributing to our understanding of the molecular basis of plant diversity and enabling genetic improvements. However, with the extensive application of genomics at the population-level and in interspecies research, the limitations of using a single reference genome have become increasingly evident. For instance, it is challenging to use a single reference genome for capturing rare and complex genomic regions within a population, which often serve as important sources of plant genetic diversity (Wang et al., 2023c). To address these limitations, pangenomes that represent all genomic information of an entire population or taxa have gained prominence over the past decade (Shi et al., 2023). By expanding the scope of genomics to the genus level, this concept has further evolved into super-pangenomes (Khan et al., 2020). Super-pangenomes have established a crucial foundation for the protection and utilization of plant germplasm resources on a larger scale, particularly through the incorporation of wild relatives and molecular breeding for cultivated plants, emerging as a key research frontier in genomics. Here, we explore the conceptual evolution, construction strategies, and research achievements of plant super-pangenomes, providing a panoramic perspective and valuable insight for the effective use and better understanding of this powerful tool.

## Development of the concepts of the pangenome and super-pangenome in plants

High-quality genome resources form the essential foundation for research areas such as germplasm conservation, utilization, and innovation in plants (Garg et al., 2024). As more high-quality reference genomes become available, the genomes of representative or key samples across diverse plant species have been analyzed and employed to discover valuable genetic variations and underlying molecular mechanisms. When comparing genomic differences among accessions within a population, it is evident that numerous complex structural variations exist, which cannot be fully captured by a single reference genome (Cheung et al., 2009). Therefore, to achieve a more comprehensive understanding of population diversity and to reduce the influence of reference genome bias on the detection of genetic variations, the pangenome concept, derived from bacterial research (Tettelin et al., 2005), has gained widespread attention. A pangenome represents the sum of all genomic information within a species. This concept has been successfully applied in human (Li et al., 2010) and *Arabidopsis* (Cao et al., 2011) populations and has been further improved and expanded through long-term studies of pangenomes in numerous animal and plant species (Eisenstein, 2023). A widely recognized pangenome usually consists of three components: (1) the core genome, present within all individuals of that population, essential for critical life processes; (2) the dispensable genome, found in two or more individuals, often associated with environmental adaptability; and (3) the private genome, present only in a single individual, often related to rare traits or phenotypes in the population and constitutes a significant rare gene pool.

**Figure 1:**
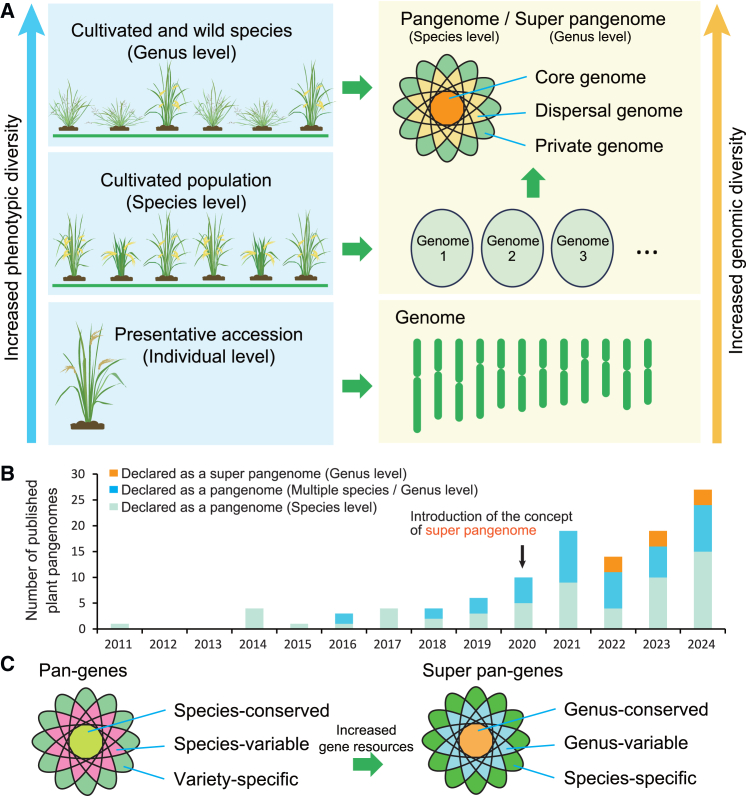
The development of the plant super-pangenome. **(A)** Illustration of the evolutionary progression from genome to super-pangenome. **(B)** Timeline of published plant pangenomes and super-pangenomes. Statistical data were obtained from PubMed records with Medical Subject Headings (MeSH) terms limited to “genome∗” or “genomic∗”; the cutoff date was October 11, 2024. **(C)** Compositional disparities between pangenomes and super-pangenomes.

For decades, extensive pangenomic research has been conducted to investigate the population diversity of different plant species, particularly the diversity of cultivated germplasms accumulated over the centuries (Schreiber et al., 2024). With the inclusion of more wild relatives into the pangenome panel, the super-pangenome has become a more comprehensive framework to capture richer genomic and phenotypic diversity at the genus level in plants (Figure 1A). Originally designed as a pangenome of multiple pangenomes from different populations or species (Khan et al., 2020), most super-pangenomes are in practice directly constructed from the genomes of accessions representing different species. This has led to some ambiguity in distinguishing between pangenomes and super-pangenomes. Since the initial release of the *Arabidopsis thaliana* pangenome (Cao et al., 2011), over 110 pangenomes or super-pangenomes have been assembled for plant species, with at least 53 having been constructed using two or more species. However, only nine were explicitly declared as super-pangenomes (Figure 1B). Nevertheless, compared to a typical species-level pangenome, the widely recognized super-pangenome generally encompasses a broader range of genetic resources, including all genes from multiple representative species sampled at the genus level (super-pangenes), usually comprising genus-conserved (core genome), genus-variable (dispensable genome), and species-specific (private genome) gene sets (Figure 1C). Therefore, the plant super-pangenome represents an expansion of the pangenome, covering the genomic information of all concerned species, particularly the abundant genomic variations found in wild species. The super-pangenome possesses significant potential for practical application. In crops, super-pangenome research typically integrates cultivated varieties and their wild relatives for the exploitation and utilization of wild alleles in crop improvement.

## Discrimination between the plant pangenome and super-pangenome

Although there is currently a lack of a clear distinction between pangenomes and super-pangenomes, discussing the differences between the two is conducive to a better understanding of the super-pangenome concept. First, the sampling scopes differ. Typical pangenome research covers different populations within a single species, while super-pangenome research can sample at the genus level or even higher taxonomic levels, such as the family level or above, thereby encompassing multiple species. Second, the dataset compositions differ. A pangenome dataset contains information on sequences, genes, and variants from all genomes within a species, whereas a super-pangenome dataset includes genomic information on sequences, genes, and variants from multiple species. When the sampling scope is large enough, the super-pangenome can also include the pangenomes of all the sampled species, effectively functioning as a super-collection of multiple pangenomes derived from different species (Khan et al., 2020). Finally, their implications differ. A pangenome represents the total genomic information of a single species, reflecting its genetic diversity. In contrast, a super-pangenome represents the genomic information of multiple species, reflecting the diversity of plant genes and functional variations at the genus level or higher. To provide a comprehensive review of the plant super-pangenome and its applications, we classify any pangenome research covering two or more species (regardless of prior declaration as a super-pangenome) as super-pangenome research.

## Strategies for building the plant super-pangenome

**Figure 2:**
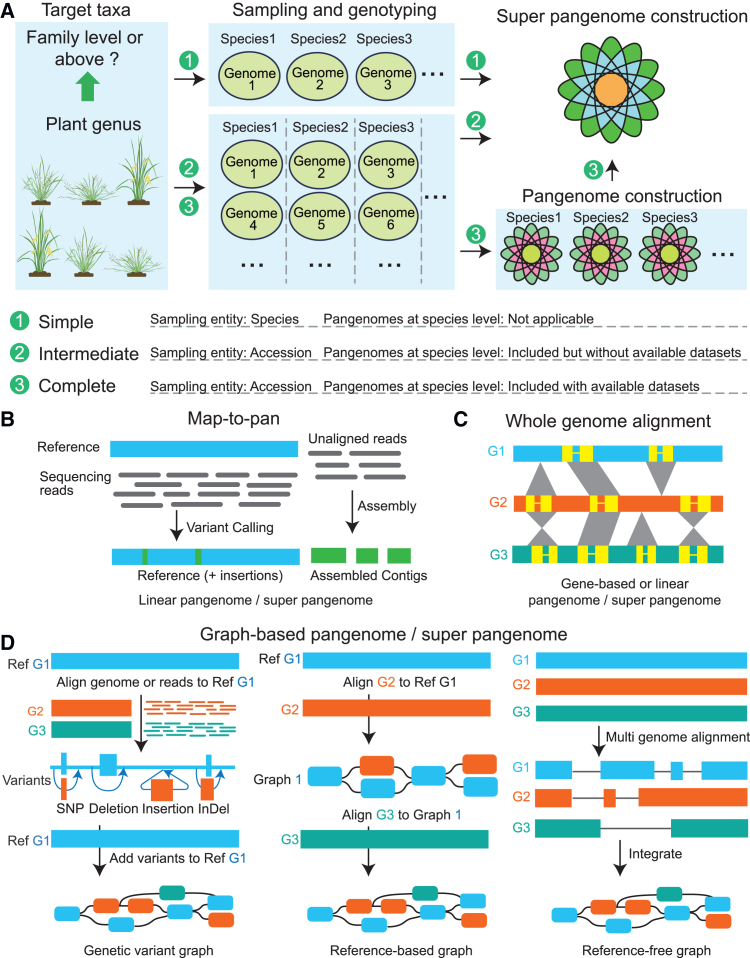
Pipelines for constructing plant super-pangenomes using different methods. **(A)** Comprehensive flowchart depicting three pipelines for building a super-pangenome. **(B–D)** Illustrations of pangenome or super-pangenome construction based on map to pan **(B)**, whole-genome alignment **(C),** and graph-based **(D)** strategies. G: genome. The graphical models of these algorithm processes were adapted from Rakocevic et al. (2019) and Li et al. (2020).

Based on variations in sampling scope and dataset composition, current pipelines for building plant super-pangenomes can be classified into three types (Figure 2A). (1) The simple super-pangenome, which is sampled at the species level, where only one accession is taken for each species and is constructed using conventional pangenome methods. While the simple super-pangenome can reflect the genomic diversity of plant species at the genus or higher taxonomic levels, it is not a collection of multiple pangenomes from those species. (2) The intermediate super-pangenome, which is sampled at the accession level, where some species include multiple accessions, and is constructed by conventional pangenome methods. Although the intermediate super-pangenome could theoretically include pangenome information on multiple species, it does not include specifically defined datasets as seen in the original definition of the super-pangenome (Khan et al., 2020), and thus we term it the intermediate super pangenome. (3) The complete super-pangenome, where each species’ pangenomes are first constructed, and then a multi-species super-pangenome is generated by integrating them. This process is relatively complex but simultaneously incorporates the genomic information of the target taxa and the pangenomes of sampled species. To date, published plant super-pangenomes mainly consist of simple and intermediate, with only a few complete super-pangenomes constructed (Table 1).

**Table 1:** Summary of super-pangenome studies in plants

Genus	Crop	Sequencing platform	Construction methods	*N*_species_	*N*_accession_	References
*Actinidia*	kiwifruit	Illumina; Nanopore	simple/whole-genome alignment	9	9	Li et al. (2024b)
*Brachypodium*	non-crop	Illumina; PacBio	intermediate/whole-genome alignment	3	56	Gordon et al. (2020)
*Brassica*	oilseed rape, mustard rape, cabbage, cauliflower, etc.	Illumina	intermediate/map to pan	2	10	Golicz et al. (2016)
–	–	Illumina	complete/map to pan and whole-genome alignment	3	243	Bayer et al. (2021)
–	–	PacBio/Nanopore; Illumina	intermediate/whole-genome alignment	6	15	He et al. (2021)
*Capsicum*	pepper	Illumina	intermediate/map to pan	4	383	Ou et al. (2018)
*Cicer*	chickpea	Illumina; Hi-C	simple/whole-genome alignment and graph based	10	10	Khan et al. (2024)
–	–	Illumina	intermediate/map to pan	2	3366	Varshney et al. (2021)
*Citrullus*	watermelon	Illumina; PacBio; Nanopore; Hi-C	intermediate/whole-genome alignment and graph based	7	27	Zhang et al. (2024b)
–	–	Illumina; PacBio	complete/map to pan and whole-genome alignment	4	547	Wu et al. (2023b)
–	–	Illumina	intermediate/whole-genome alignment	7	400	Sun et al. (2023)
*Citrus*	orange, etc.	public data	intermediate/whole-genome alignment	17	22	Liu et al. (2022)
*Cochlearia*	non-crop	PacBio/Nanopore; Illumina	intermediate/graph based	8	24	Hämälä et al. (2024)
*Fragaria*	strawberry	PacBio; Nanopore	simple/graph based	6	6	Qiao et al. (2021)
*Glycine*	soybean	Illumina; PacBio; Hi-C	intermediate/whole-genome alignment	7	31	Zhuang et al. (2022)
–	–	PacBio	intermediate/graph based	3	27	Liu et al. (2020)
–	–	Illumina	intermediate/map to pan	5	1110	Bayer et al. (2022)
*Gossypium*	cotton	PacBio/Nanopore; Illumina	intermediate/map to pan	2	344	He et al. (2024c)
–	–	Illumina	intermediate/map to pan	2	1961	Li et al. (2021)
–	–	Illumina	intermediate/graph based	5	11	Jin et al. (2023)
–	–	public dataset	simple/whole-genome alignment	26	27	Song et al. (2024)
–	–	Illumina; Nanopore; Hi-C	whole-genome alignment	9	10	Wang et al. (2022)
*Hevea*	rubber tree	Nanopore	intermediate/whole-genome alignment	4	8	Fang et al. (2024)
*Hordeum*	barley	Illumina	intermediate/map to pan	2	12 306	Gao et al. (2020)
*Malus*	apple	Nanopore	intermediate/whole-genome alignment	2	7	Su et al. (2024)
–	–	Illumina; 10× Genomics	intermediate/whole-genome alignment	3	93	Sun et al. (2020)
*Musa*	banana	Illumina	intermediate/map to pan	10	15	Rijzaani et al. (2022)
*Oryza*	rice	Illumina; Nanopore; Hi-C	intermediate/whole-genome alignment and graph based	4	251	Shang et al. (2022)
–	–	PacBio	intermediate/graph based	2	33	Qin et al. (2021)
–	–	PacBio	intermediate/whole-genome alignment	2	74	Wu et al. (2023a)
–	–	Illumina	intermediate/whole-genome alignment	2	66	Zhao et al. (2018b)
–	–	public dataset	intermediate/whole-genome alignment	2	12	Wang et al. (2023a)
–	–	Illumina; Nanopore	intermediate/whole-genome alignment	2	111	Zhang et al. (2022a)
*Pisum*	pea	Illumina	intermediate/whole-genome alignment	3	116	Yang et al. (2022)
*Populus*	poplar	Illumina; Nanopore; Hi-C	simple/whole-genome alignment	18	19	Shi et al. (2024)
–	–	Illumina	intermediate/map to pan	3	7	Pinosio et al. (2016)
–	–	Illumina	simple/map to pan	10	10	Zhang et al. (2019)
*Sesamum*	sesame	Illumina; PacBio; Roche/454; Hi-C	simple/whole-genome alignment	7	7	Miao et al. (2024)
*Setaria*	millet	PacBio; Illumina	intermediate/graph based	3	112	He et al. (2023)
*Solanum*	eggplant	Illumina	intermediate/whole-genome alignment	3	25	Barchi et al. (2021)
–	–	Illumina	intermediate/whole-genome alignment	3	67	Song et al. (2019)
–	potato	Illumina	intermediate/whole-genome alignment	60	296	Bozan et al. (2023)
–	tomato	PacBio; Hi-C; Bionano	intermediate/whole-genome alignment and graph based	11	13	Li et al. (2023)
–	–	PacBio/Nanopore; Illumina	intermediate/graph based	3	838	Zhou et al. (2022)
–	–	Illumina	intermediate/map to pan	4	725	Gao et al. (2019)
*Sorghum*	sorghum	PacBio	intermediate/graph based	4	16	Tao et al. (2021)
*Triticeae*	wheat, barley	PacBio	intermediate/whole-genome alignment	15	67	Chen et al. (2023b)
–	–	Illumina	intermediate/whole-genome alignment	4	15	Chen et al. (2020)
*Vitis*	grape	PacBio; Bionano	simple/graph based	9	9	Cochetel et al. (2023)
–	–	PacBio; Nanopore; Hi-C	intermediate/graph based	5	18	Liu et al. (2024)
*Zea*	maize	public dataset	intermediate/whole-genome alignment	8	732	Gui et al. (2022)

Constructing a super-pangenome based on individual genomes or pangenomes generally follows the same bioinformatics strategy as conventional pangenome construction. Thus, their construction methods are discussed together here. To date, there are three primary strategies for the construction of plant super-pangenomes: map to pan, whole-genome alignment, and graph-based pangenome (Figures 2B–2D).

Map to pan is a simplified method designed to improve the efficiency of super-pangenome construction using an available reference genome. It sequentially maps the genomic sequencing reads of different samples to the reference genome, assembles the non-mapped reads into contigs representing a mixture of all non-reference sequences, and integrates these contigs with the reference genome to generate a linear super-pangenome with non-redundant sequences (Figure 2B). This strategy is cost-effective because each sample can be sequenced at a relatively low sequencing depth, which allows the pooling of hundreds or even thousands of samples. It is capable of gradually expanding the coverage of the genome, especially for species with complex genomes. However, its effectiveness heavily depends on the completeness and accuracy of the reference genome. As there is no assembly process, the map-to-pan method struggles with repeat-rich genomes and cannot detect complex or large structural variants (SVs) that cannot be covered by single short reads.

The whole-genome alignment strategy is the most straightforward way to construct a super-pangenome (Figure 2C). It involves assembling a high-quality individual genome using short and long sequencing reads, followed by comparative analyses to generate a super-pangenome that includes all non-redundant genes and/or genomic variants (Li et al., 2022). Currently, whole-genome alignment is the most widely used method, which does not rely on a reference genome and directly reflects the genetic variation among samples. However, assembling high-quality genomes for plant species with large, highly repetitive, heterozygous, and polyploid genomes remains challenging. Constructing a super-pangenome with large-scale samples is costly (Hurgobin and Edwards, 2017).

The graph-based strategy is the most promising approach for constructing a complete super-pangenome, as it stores all genomic sequences in a graph format that can be used as a reference for SNP and SV detection using both sequencing reads and assemblies (Wang et al., 2023b). It has recently emerged as a significant alternative to the traditional linear super-pangenome. It combines all genetic variants in a population, ensuring sequence continuity and accurately displaying the topology of SVs among different samples, offering advantages over conventional linear pangenomes. Currently, there are three approaches for generating a graph-based super-pangenome: the genetic variation graph, the reference-based graph, and the reference-free graph (Figure 2D).

To generate a genetic variation graph, genomic variants are identified for a population based on a reference genome, and then these variants are added to the reference genome. Tools such as the variation graph toolkit (Garrison et al., 2018) and Graphtyper2 (Eggertsson et al., 2017) are available to support this approach. A genomic variation graph can incorporate SNPs, insertions or deletions (indels), and SVs (>50 bp). Although this is a relatively simple approach, it is highly dependent on the quality of the reference genome and the variation calling process, making it insufficient for characterizing complex and large SVs (Wang et al., 2023b).

To generate a reference-based graph, the genomes of a population are sequentially aligned to a reference genome or the resulting pangenome graph, incrementally augmenting the graph with long query subsequences. This method, supported by tools like minigraph (Li et al., 2020) or minigraph-cactus (Hickey et al., 2024), is much faster than other methods and can effectively identify and integrate large SVs. However, it is less effective with SNPs and may introduce bias, potentially overlooking genetic variations exclusive to non-reference genomes. Additionally, the selection of different reference genomes and the order of alignment can result in varying pangenome graphs for the same population.

The reference-free graph, as suggested by its name, does not require a reference genome. To construct this graph, multiple genomes are aligned pairwise and divided into different aligned fragments, which are then clustered and converted into nodes on the graph. Supported by tools like PGGB (Garrison et al., 2024) and Cactus (Armstrong et al., 2020), this method ensures that the pangenome graph captures genomic diversity without reference bias by treating all genomes equally. However, it requires significant computational resources and complicates downstream analysis, particularly for large genomes with extensive repeat regions. Also, the optimal parameters for the PGGB tool vary across different species, typically requiring multiple computational experiments to fine-tune.

## Progress and achievements in plant super-pangenomic research

High-quality pangenomes are highly dependent on species/population diversity and the quality/quantity of accumulated genomic data. As a result, pangenome research has primarily focused on food and economically important crops, as these taxa have amassed extensive germplasm and genomic data resources over the past few decades (Sun et al., 2022; Shi et al., 2023), thereby providing more mature conditions for conducting pangenome research. In 2014, through third-generation sequencing technology, plant pangenomics entered a golden era, rapidly achieving widespread application across various plant species. Since 2016, pangenomics has evolved toward multi-species coverage and comparisons, culminating in the emergence of the super-pangenome (Figure 1B). To date, nearly all super-pangenomes (including pangenomes covering multiple species) are derived from crop species and their closely related wild species (Table 1). More than 25 crops have super-pangenomes for their respective genera (Table 1), significantly expanding pangenomic sequences and pangene resources across diverse germplasms, particularly from wild relatives. For example, the graph-based rice super-pangenome, constructed from 251 high-quality genomes of two cultivated rice species and their wild relatives, contained a total of 1.52 Gb non-redundant DNA sequences, which is approximately four times larger than an individual rice genome. It includes 1.15 Gb sequences and ∼12 000 genes that are absent from the Nipponbare reference genome (Kawahara et al., 2013), most of which were contributed by the wild rice species (Shang et al., 2022). In *Citrullus*, T2T assemblies of 27 genotypes from seven *Citrullus* species were integrated to generate a T2T super-pangenome, approximately 1.5 times larger than any individual genome, adding 399.2 Mb and 11 225 genes compared to the cultivated watermelon, with more than half derived from wild relatives (Zhang et al., 2024b). The *Populus* super-pangenome, covering 19 accessions from 18 species, contains 712 487 genes, of which an average of 51.3% per accession belong to the core gene set (Shi et al., 2024). These super-pangenome resources offer valuable genetic resources and powerful tools for critical research areas such as crop domestication, the exploration and utilization of favorable genes, molecular breeding, evolutionary adaptability, and polyploidization in plants.

To meet diverse research demands and leverage accumulated sequencing data, super-pangenome datasets constructed by different studies vary in taxonomic composition, content, and variation resources. To comprehensively explore genetic variations in thousands of chickpea (*Cicer arietinum*) accessions and identify wild sources of genetic superiority, the chickpea pangenome was constructed using a map-to-pan approach to maximize the integration of available data, including genomic sequencing data and *de*-*novo-*assembled sequences derived from up to 3171 cultivated (*C. arietinum*) and 195 wild (*Cicer reticulatum*) accessions (Varshney et al., 2021). This linear pangenome, which included 592.58 Mb genomic sequences and 29 870 genes (1582 novel gene models), was successfully used to identify domestication and migration signatures, superior haplotypes, and deleterious mutations for chickpea breeding. Many domesticated crops tend to have narrow genetic diversity due to long-term artificial selection, exhibiting a bottleneck in their genetic improvement. Utilizing wild relatives is key to overcoming such challenges. For example, a tomato super-pangenome was constructed from 11 wild and cultivated species in the *Solanum* section *Lycopersicon* (the tomato clade), incorporating 13 chromosome-level reference genomes and yielding 40 457 pangene families (Li et al., 2023). Of these, only 23 839 (54.0%) were conserved core genes, fewer than in a previously reported tomato pangenome of 586 accessions from two wild and cultivated species (Gao et al., 2019). A graph-based super-pangenome was also generated using these 13 genomes and an additional 100 tomato genomes to enable SV calling in a large-scale population and the SV-based genome-wide association studies (Li et al., 2023). To constructed a phylogenomic framework for examining the evolutionary history of strawberry (*Fragaria* spp.) species, alignments of the genomes from six *Fragaria* species were conducted to generate a pangenome for *Fragaria*, revealing more than 25 000 pangenes, 42.9% of which were core genes. This framework provides an important foundation for elucidating the evolutionary dynamics of gene families *in Fragaria* (Qiao et al., 2021).

To maximize the potential of these pangenome resources, many super-pangenome studies have established online databases to provide open and user-friendly access to the scientific community. Currently, at least 11 crop species, including rice (Zhao et al., 2018a; Shang et al., 2022), wheat (Chen et al., 2020), soybean (Bayer et al., 2022; Zhuang et al., 2022), rapeseed (Bayer et al., 2021), tomato (Li et al., 2023), watermelon (Zhang et al., 2024b), citrus (Liu et al., 2022), strawberry (Qiao et al., 2021), grape (Cochetel et al., 2023), and poplar (Shi et al., 2024), have online databases based on their super-pangenome datasets. These platforms feature tools for pangenome browsing, dataset querying, co-linearity and structural variation visualization, and multi-omics data analyses. For example, the Rice Super Pan-genome Information Resource Database (http://www.ricesuperpir.com/) allows for the visual exploration of genome-wide collinearity and variations among 251 genomes in four wild and cultivated rice species (Shang et al., 2022), providing high-quality assemblies and gene annotations. This is a highly efficient and powerful research tool for fully exploiting genomic diversity and conducting functional genomics studies in wild and cultivated rice. The SoyBase website (http://www.soybase.org/) provides sequence variation maps, pangene sets, BLAST services, and downloadable datasets for *Glycine max and wild Glycine species* (Zhuang et al., 2022). The Citrus Pan-genome to Breeding Database (http://citrus.hzau.edu.cn/) includes 23 genomes from 17 citrus species, featuring orthologous genes, functional annotations, and multi-omics datasets, such as gene expression and DNA methylome datasets for different wild species (Liu et al., 2022). These online resources greatly enhance the utility of super-pangenomes in studying the evolution, domestication, and breeding of crops and their wild relatives.

## Major applications of plant super-pangenomes

**Figure 3:**
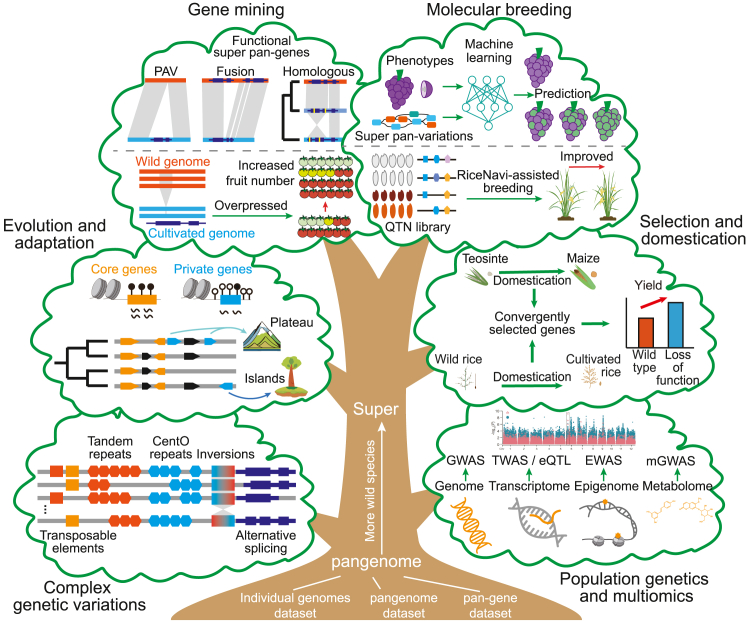
Key applications of super-pangenomes in plants. The major components of the plant super-pangenome datasets and their representative applications across genomics, population genetics, evolutionary biology, functional genomics, crop domestication, and breeding.

Covering multiple wild and cultivated species, super-pangenomes provide an important foundation for research areas such as genomic variation dynamics, population genetics, evolution, domestication, gene mining, and crop breeding in different plant species (Figure 3). Here, we summarize the achieved and potential applications of plant super-pangenomes to offer a comprehensive perspective and a better understanding of the developmental requirements and limitations of super-pangenomic research.

### Complex genetic variations

Genomic variation is a crucial foundation for plant phenotypic diversity and heritability. Conventional variation detection methods typically identify small sequence variations in homologous regions by mapping genomic sequencing reads to a single reference genome. However, it is often challenging to detect variations in highly repetitive, structurally complex, and low-homology regions due to the low efficiency and accuracy of read mapping (Bayer et al., 2020). The super-pangenome, encompassing the complete genomic sequences and gene data of multiple species, facilitates direct comparisons of genomic sequences with various complex regions and repetitive elements. Moreover, graph-based pangenomes have demonstrated advantages in aligning short sequences to complex SV regions and achieving more accurate genotyping of SVs (Wang et al., 2023b). These improvements allow for comprehensive exploration of previously undetected, intricate genetic variations and aid in recovering the "missing heritability" in crop research (Zhou et al., 2022). By leveraging super-pangenome datasets, various complex SVs, such as inversions (Zhou et al., 2023; He et al., 2024b), tandem repeat sequences (He et al., 2024a), centromere region variations (Lv et al., 2024), transposon variations (Li et al., 2024c), and alternative splicing (Zhang et al., 2024a) of functional genes, have been effectively identified across different populations or species. These discoveries have significantly expanded the available genetic variation resources and provide a crucial foundation for critical research fields such as plant genetics, evolutionary biology, and crop breeding.

### Population genetics and multi-omics

To optimize the use of available germplasm resources from closely related wild species for crops, an increasing number of population genetics studies have begun incorporating samples from both cultivated and wild species. This approach expands the diversity of the population and the potential beneficial gene pool (Schreiber et al., 2024). By covering dispensable and private genomic regions from different species, the super-pangenome circumvents the bias associated with a single reference genome and reveals more comprehensive genotype information, particularly multi-allelic genotypes, across large-scale populations comprised of multiple species (Raza et al., 2023). Consequently, the super-pangenome has emerged as an ideal tool for population genetics research. To date, most plant super-pangenomes have been directly or indirectly used to construct population-level genomic variation maps for population genetics analyses, such as SV-based genome-wide association studies, aimed at identifying significant functional genes related to economic traits and ecological adaptations. For example, a 1.4 kb deletion identified using the rice super-pangenome was found to be associated with grain yield, a variation that could not be detected through conventional SNP analyses based on a single linear reference genome (Shang et al., 2022). In recent years, the continuous accumulation of multi-omics data has further enriched the genotypic and phenotypic datasets of plant populations. This has enabled the super-pangenome to be widely applied in identifying superior variations at the transcriptional, epigenetic, and metabolic levels (Wei et al., 2024a; Ferrero-Serrano et al., 2024; Shi et al., 2024), significantly expanding the application of super-pangenomic datasets in population genetics and demonstrating their rich scalability and potential.

### Evolution and adaptation

Unlike typical species-level pangenomes, super-pangenomes are capable of identifying conserved, variable, and private genes at the genus level across multiple species (Figure 1C), providing crucial evidence for studying species divergence and its correlation with evolutionary drivers, such as environmental adaptability. For example, leveraging genomic data from 19 *Populus* species, a super-pangenome of the genus *Populus* was constructed, encompassing core (51.11%), semi-conserved (22.78%), dispensable (22.50%), and species-specific (3.61%) genes (Shi et al., 2024). Compared to other gene categories, species-specific genes demonstrated higher levels of methylation modification and lower levels of gene expression, and played key roles in species adaptation to diverse climates, such as temperate plateaus and tropical islands. Similarly, using high-quality genomes from diploid and allopolyploid species, a super-pangenome of the genus *Glycine* was constructed to explore the biased subgenomic fractionation during polyploidization. This analysis identified two genes associated with vegetative-reproductive phase transition and lateral shoot outgrowth, which may have contributed to the perenniality-annuality transition in this genus (Cochetel et al., 2023). These studies highlight the substantial advantages of super-pangenomes in addressing evolutionary biological questions at the genus level and above.

### Selection and domestication

From wild ancestors to domesticated plants, the process of domestication involves extensive artificial selection of wild traits. Uncovering the genetic basis and molecular mechanisms behind crop domestication is an important approach to understanding the formation of important agronomic traits (Huang et al., 2022). Since most high-quality reference genomes are derived from cultivated species, genetic variation maps that rely on a single reference genome often have difficulty completely covering the regions involved in the domestication process. Super-pangenomes provide a powerful solution to this bottleneck by comprehensively evaluating the genetic diversity of populations across multiple species. For instance, using a pangenomic dataset of wild and cultivated accessions, 490 orthologous genes were identified as convergently selected in the domestications of rice and maize. Evolutionary and functional evidence supported the convergent selection of KRN2/OsKRN2 for grain number in both crops, with their complete loss-of-function alleles contributing to increased grain yield (Chen et al., 2022). Similarly, a pangenome-wide analysis of the LONELY GUY cytokinin biosynthesis gene family across the *Solanum* genus and other flowering plants revealed that LONELY GUY mutations were associated with convergent prickle losses in both cultivated and wild species, as well as outgroup species in flowering plants (Satterlee et al., 2024). Additionally, super-pangenome datasets generated from wild and cultivated populations enable the construction of whole-genome variation maps at the genus level. These maps provide landscape analyses of selective sweeps and domestication-related genes (Gui et al., 2022; Wu et al., 2023b), offering a macroscopic perspective on the genetic basis of crop domestication.

### Gene mining

The efficient exploration and utilization of ecologically and economically favorable genes constitute an important basis for research on plant evolution and crop genetic improvement (Fernie and Yan, 2019). The plant super-pangenome captures the rich diversity of various species at the genus level, showcasing a wealth of gene resources from both wild and cultivated genotypes (Liu et al., 2020). This serves as an essential foundational platform and efficient tool for gene-mining research in plants. For example, by applying a graph-based pangenome constructed from 11 cotton accessions across five allotetraploid species, researchers identified genomic SVs responsible for speciation, domestication, and improvement in tetraploid cotton, including a 9-bp indel associated with the elimination of interspecific reproductive isolation between *Gossypium hirsutum* and *Gossypium barbadense* (Jin et al., 2023). Similarly, a super-pangenome analysis of the *Solanum* section *Lycopersicon* enabled the construction of a tomato-clade-wide gene repertoire, leading to the discovery of a wild tomato gene with the potential to increase the yield of modern cultivated tomatoes, as well as numerous quantitative trait loci associated with tomato-flavor-related traits and fruit metabolites (Li et al., 2023). Recently, a pangenome consisting of 16 founders for a permanent population of 18 421 (18 000) rice lines was used to identify 96 high-quality candidate genes and 170 masking epistasis quantitative trait locus pairs related to 16 traits (Wei et al., 2024b). By integrating more diverse germplasm from different species, super-pangenomes offer significant promise for further unlocking the potential to mine more favorable genes in the future.

### Molecular breeding

Wild species often contribute greater sequence diversity to crop super-pangenomes, especially wild alleles that have been lost in cultivated varieties due to genetic bottlenecks during the domestication process and could play a crucial role in regulating economic traits in crops (Qiao et al., 2021; Li et al., 2023; Zhang et al., 2024b). Conducting in-depth scans of genomes across large-scale taxa at the genus or family level, including wild species, allows for the identification of comprehensive genomic selective loci, including evolutionary constraints and deleterious mutations. This information can be efficiently applied to assess the breeding potential of germplasm and provide crucial guidance for addressing breeding challenges (Wu et al., 2023c). For example, by constructing a comprehensive map of quantitative trait nucleotides for different rice germplasm resources, a genome navigation system called RiceNavi was developed. This system facilitates the pyramiding of quantitative trait nucleotides and optimizes breeding routes for rice improvement (Wei et al., 2021). Furthermore, high-quality genomic data from large-scale populations can be applied to build genomic selection models based on advanced algorithms and prediction models for crop phenotypes, which hold promise for the development of smart crop breeding (Li et al., 2024a; Ferrero-Serrano et al., 2024; Liu et al., 2024). Such applications demonstrate great potential for linking super-pangenomic knowledge to diverse crop improvement needs.

## Challenges and prospects

Super-pangenomes have revolutionized the study of plant genetics by providing insight into the genetic basis of key traits and facilitating the conservation and utilization of plant germplasm resources. These advances highlight the unique advantages and great potential for practical application. However, unlike plant genomics, which has extensively explored agricultural, medicinal, ornamental, and protected plant species across both angiosperms and gymnosperms (Sun et al., 2022), research on plant pangenomes and super-pangenomes remains predominantly concentrated on significant agricultural crops within angiosperms and their related species (Table 1). This is likely due to the long-term and systematic studies conducted on these crops, which have resulted in the accumulation of representative populations, high-quality genomic sequencing data, and genome assemblies, and corresponding phenotypic datasets, thereby fulfilling the essential requirements for super-pangenome research. As plant genomics continues to include more species, it will facilitate the widespread application of pangenomics and super-pangenomics research, potentially on a larger taxonomic scale (e.g., family level or above), providing powerful tools for addressing diverse challenges in downstream research fields.

High heterozygosity, repetition, and polyploidy are highly prevalent in plant genomes (Sun et al., 2022). Effectively integrating and handling large-scale complex sequence information to efficiently cover genomic data from different species remains a significant challenge in advancing plant super-pangenomes. The commonly used super-pangenome construction methods, which add non-reference sequences to a reference genome to form a linear sequence collection (Hu et al., 2020; Zhang et al., 2022b), cannot fully capture sequence deletions, inversions, translocations, and multi-allelic variations. While graph-based super-pangenomes are capable of effectively capturing genetic variations and, therefore, represent the future of reference genomes, storage and visualization remain major technical challenges (Hübner, 2022). For example, coordinate restoration for graph-based assemblies is complex, and it is currently difficult to accurately represent the original coordinates of target sequences in donor genomes. Most bioinformatics analysis tools are designed for linear reference genomes, and the supporting tools for graph-based reference genomes remain limited (Wang et al., 2023b). Additionally, the lack of a unified quality assessment standard is a common challenge for pangenome and super-pangenome studies. Addressing these problems will significantly enhance the development of super-pangenomes.

Future super-pangenome research could focus on building a scalable coordinate system and developing a more comprehensive suite of supporting analysis tools to better accommodate evolving application demands. These demands may include using gene expression profiles from diverse tissues under different environmental conditions to generate complete gene annotations; constructing more comprehensive genomic variation maps to explore phenotypic heritability; performing integrated analyses with multi-omics data, such as the epigenome, metabolome, and proteome, to uncover the full genetic basis of economically important traits; expanding the range of plant species studied to elucidate the evolutionary and ecological adaptation mechanisms across larger-scale plant taxa; and integrating intelligent algorithm frameworks, such as machine learning, to enhance gene mining and advanced crop breeding. In summary, the development of plant super-pangenomes not only deepens our theoretical understanding of plant genetic diversity and evolutionary processes but also provides practical tools for the conservation, utilization, and genetic improvement of plant germplasm resources.

## Funding

This work was funded by the 10.13039/501100001809National Natural Science Foundation of China (32188102 and 32372148), the Innovation Program of Chinese Academy of Agricultural Sciences (CAAS-CSIAF-202303), and the 10.13039/501100012166National Key R&D Program of China (2022YFE0139400).

## Acknowledgments

No conflict of interest declared.

## Author contributions

W.H. and X.L. wrote the manuscript. L.S. and Q.Q. revised the manuscript.

## References

[bib1] Arabidopsis Genome Initiative (2000). Analysis of the genome sequence of the flowering plant *Arabidopsis thaliana*. Nature.

[bib2] Armstrong J., Hickey G., Diekhans M., Fiddes I.T., Novak A.M., Deran A., Fang Q., Xie D., Feng S., Stiller J. (2020). Progressive cactus is a multiple-genome aligner for the thousand-genome era. Nature.

[bib3] Barchi L., Rabanus-Wallace M.T., Prohens J., Toppino L., Padmarasu S., Portis E., Rotino G.L., Stein N., Lanteri S., Giuliano G. (2021). Improved genome assembly and pan-genome provide key insights into eggplant domestication and breeding. Plant J..

[bib4] Bayer P.E., Golicz A.A., Scheben A., Batley J., Edwards D. (2020). Plant pan-genomes are the new reference. Nat. Plants.

[bib5] Bayer P.E., Valliyodan B., Hu H., Marsh J.I., Yuan Y., Vuong T.D., Patil G., Song Q., Batley J., Varshney R.K. (2022). Sequencing the USDA core soybean collection reveals gene loss during domestication and breeding. Plant Genome.

[bib6] Bayer P.E., Scheben A., Golicz A.A., Yuan Y., Faure S., Lee H., Chawla H.S., Anderson R., Bancroft I., Raman H. (2021). Modelling of gene loss propensity in the pangenomes of three *Brassica* species suggests different mechanisms between polyploids and diploids. Plant Biotechnol. J..

[bib7] Bozan I., Achakkagari S.R., Anglin N.L., Ellis D., Tai H.H., Strömvik M.V. (2023). Pangenome analyses reveal impact of transposable elements and ploidy on the evolution of potato species. Proc. Natl. Acad. Sci. USA.

[bib8] Cao J., Schneeberger K., Ossowski S., Günther T., Bender S., Fitz J., Koenig D., Lanz C., Stegle O., Lippert C. (2011). Whole-genome sequencing of multiple *Arabidopsis thaliana* populations. Nat. Genet..

[bib9] Chen J., Wang Z., Tan K., Huang W., Shi J., Li T., Hu J., Wang K., Wang C., Xin B. (2023). A complete telomere-to-telomere assembly of the maize genome. Nat. Genet..

[bib10] Chen W., Chen L., Zhang X., Yang N., Guo J., Wang M., Ji S., Zhao X., Yin P., Cai L. (2022). Convergent selection of a WD40 protein that enhances grain yield in maize and rice. Science.

[bib11] Chen Y., Song W., Xie X., Wang Z., Guan P., Peng H., Jiao Y., Ni Z., Sun Q., Guo W. (2020). A collinearity-incorporating homology inference strategy for connecting emerging assemblies in the triticeae tribe as a pilot practice in the plant pangenomic era. Mol. Plant.

[bib12] Chen Y., Guo Y., Xie X., Wang Z., Miao L., Yang Z., Jiao Y., Xie C., Liu J., Hu Z. (2023). Pangenome-based trajectories of intracellular gene transfers in Poaceae unveil high cumulation in Triticeae. Plant Physiol..

[bib13] Cheung F., Trick M., Drou N., Lim Y.P., Park J.-Y., Kwon S.-J., Kim J.-A., Scott R., Pires J.C., Paterson A.H. (2009). Comparative analysis between homoeologous genome segments of *Brassica napus* and its progenitor species reveals extensive sequence-level divergence. Plant Cell.

[bib14] Cochetel N., Minio A., Guarracino A., Garcia J.F., Figueroa-Balderas R., Massonnet M., Kasuga T., Londo J.P., Garrison E., Gaut B.S., Cantu D. (2023). A super-pangenome of the North American wild grape species. Genome Biol..

[bib15] Eggertsson H.P., Jonsson H., Kristmundsdottir S., Hjartarson E., Kehr B., Masson G., Zink F., Hjorleifsson K.E., Jonasdottir A., Jonasdottir A. (2017). Graphtyper enables population-scale genotyping using pangenome graphs. Nat. Genet..

[bib16] Eisenstein M. (2023). Every base everywhere all at once: Pangenomics comes of age. Nature.

[bib17] Fang Y., Xiao X., Lin J., Lin Q., Wang J., Liu K., Li Z., Xing J., Liu Z., Wang B. (2024). Pan-genome and phylogenomic analyses highlight *Hevea* species delineation and rubber trait evolution. Nat. Commun..

[bib18] Fernie A.R., Yan J. (2019). De Novo Domestication: An alternative route toward new crops for the future. Mol. Plant.

[bib19] Ferrero-Serrano Á., Chakravorty D., Kirven K.J., Assmann S.M. (2024). *Oryza* CLIMtools: A genome-environment association resource reveals adaptive roles for heterotrimeric G proteins in the regulation of rice agronomic traits. Plant Commun..

[bib20] Gao L., Gonda I., Sun H., Ma Q., Bao K., Tieman D.M., Burzynski-Chang E.A., Fish T.L., Stromberg K.A., Sacks G.L. (2019). The tomato pan-genome uncovers new genes and a rare allele regulating fruit flavor. Nat. Genet..

[bib21] Gao S., Wu J., Stiller J., Zheng Z., Zhou M., Wang Y.-G., Liu C. (2020). Identifying barley pan-genome sequence anchors using genetic mapping and machine learning. Theor. Appl. Genet..

[bib22] Garg V., Bohra A., Mascher M., Spannagl M., Xu X., Bevan M.W., Bennetzen J.L., Varshney R.K. (2024). Unlocking plant genetics with telomere-to-telomere genome assemblies. Nat. Genet..

[bib23] Garrison E., Sirén J., Novak A.M., Hickey G., Eizenga J.M., Dawson E.T., Jones W., Garg S., Markello C., Lin M.F. (2018). Variation graph toolkit improves read mapping by representing genetic variation in the reference. Nat. Biotechnol..

[bib24] Garrison E., Guarracino A., Heumos S., Villani F., Bao Z., Tattini L., Hagmann J., Vorbrugg S., Marco-Sola S., Kubica C. (2024). Building pangenome graphs. bioRxiv.

[bib25] Golicz A.A., Bayer P.E., Barker G.C., Edger P.P., Kim H., Martinez P.A., Chan C.K.K., Severn-Ellis A., McCombie W.R., Parkin I.A.P. (2016). The pangenome of an agronomically important crop plant *Brassica oleracea*. Nat. Commun..

[bib26] Gordon S.P., Contreras-Moreira B., Levy J.J., Djamei A., Czedik-Eysenberg A., Tartaglio V.S., Session A., Martin J., Cartwright A., Katz A. (2020). Gradual polyploid genome evolution revealed by pan-genomic analysis of *Brachypodium hybridum* and its diploid progenitors. Nat. Commun..

[bib27] Gui S., Wei W., Jiang C., Luo J., Chen L., Wu S., Li W., Wang Y., Li S., Yang N. (2022). A pan-Zea genome map for enhancing maize improvement. Genome Biol..

[bib28] Hämälä T., Moore C., Cowan L., Carlile M., Gopaulchan D., Brandrud M.K., Birkeland S., Loose M., Kolar F., Koch M.A. (2024). Impact of whole-genome duplications on structural variant evolution in Cochlearia. Nat. Commun..

[bib29] He H., Leng Y., Cao X., Zhu Y., Li X., Yuan Q., Zhang B., He W., Wei H., Liu X. (2024). The pan-tandem repeat map highlights multiallelic variants underlying gene expression and agronomic traits in rice. Nat. Commun..

[bib30] He Q., Tang S., Zhi H., Chen J., Zhang J., Liang H., Alam O., Li H., Zhang H., Xing L. (2023). A graph-based genome and pan-genome variation of the model plant Setaria. Nat. Genet..

[bib31] He W., He H., Yuan Q., Zhang H., Li X., Wang T., Yang Y., Yang L., Yang Y., Liu X. (2024). Widespread inversions shape the genetic and phenotypic diversity in rice. Sci. Bull..

[bib32] He X., Qi Z., Liu Z., Chang X., Zhang X., Li J., Wang M. (2024). Pangenome analysis reveals transposon-driven genome evolution in cotton. BMC Biol..

[bib33] He Z., Ji R., Havlickova L., Wang L., Li Y., Lee H.T., Song J., Koh C., Yang J., Zhang M. (2021). Genome structural evolution in *Brassica* crops. Nat. Plants.

[bib34] Hickey G., Monlong J., Ebler J., Novak A.M., Eizenga J.M., Gao Y., Marschall T., Li H., Paten B., Human Pangenome Reference Consortium (2024). Pangenome graph construction from genome alignments with Minigraph-Cactus. Nat. Biotechnol..

[bib35] Hu H., Yuan Y., Bayer P.E., Fernandez C.T., Scheben A., Golicz A.A., Edwards D. (2020). Legume Pangenome construction using an iterative mapping and assembly approach. Methods Mol. Biol..

[bib36] Huang X., Huang S., Han B., Li J. (2022). The integrated genomics of crop domestication and breeding. Cell.

[bib37] Hübner S. (2022). Are we there yet? Driving the road to evolutionary graph-pangenomics. Curr. Opin. Plant Biol..

[bib38] Hurgobin B., Edwards D. (2017). SNP discovery using a pangenome: Has the single reference approach become obsolete?. Biology.

[bib39] International Rice Genome Sequencing Project (2005). The map-based sequence of the rice genome. Nature.

[bib40] Jin S., Han Z., Hu Y., Si Z., Dai F., He L., Cheng Y., Li Y., Zhao T., Fang L., Zhang T. (2023). Structural variation (SV)-based pan-genome and GWAS reveal the impacts of SVs on the speciation and diversification of allotetraploid cottons. Mol. Plant.

[bib41] Kawahara Y., de la Bastide M., Hamilton J.P., Kanamori H., McCombie W.R., Ouyang S., Schwartz D.C., Tanaka T., Wu J., Zhou S. (2013). Improvement of the *Oryza sativa* Nipponbare reference genome using next generation sequence and optical map data. Rice.

[bib42] Khan A.W., Garg V., Roorkiwal M., Golicz A.A., Edwards D., Varshney R.K. (2020). Super-Pangenome by integrating the wild side of a species for accelerated crop improvement. Trends Plant Sci..

[bib43] Khan A.W., Garg V., Sun S., Gupta S., Dudchenko O., Roorkiwal M., Chitikineni A., Bayer P.E., Shi C., Upadhyaya H.D. (2024). Cicer super-pangenome provides insights into species evolution and agronomic trait loci for crop improvement in chickpea. Nat. Genet..

[bib44] Li H., Feng X., Chu C. (2020). The design and construction of reference pangenome graphs with minigraph. Genome Biol..

[bib45] Li H., Li X., Zhang P., Feng Y., Mi J., Gao S., Sheng L., Ali M., Yang Z., Li L. (2024). Smart Breeding Platform: A web-based tool for high-throughput population genetics, phenomics, and genomic selection. Mol. Plant.

[bib46] Li J., Yuan D., Wang P., Wang Q., Sun M., Liu Z., Si H., Xu Z., Ma Y., Zhang B. (2021). Cotton pan-genome retrieves the lost sequences and genes during domestication and selection. Genome Biol..

[bib47] Li N., He Q., Wang J., Wang B., Zhao J., Huang S., Yang T., Tang Y., Yang S., Aisimutuola P. (2023). Super-pangenome analyses highlight genomic diversity and structural variation across wild and cultivated tomato species. Nat. Genet..

[bib48] Li R., Li Y., Zheng H., Luo R., Zhu H., Li Q., Qian W., Ren Y., Tian G., Li J. (2010). Building the sequence map of the human pan-genome. Nat. Biotechnol..

[bib49] Li W., Liu J., Zhang H., Liu Z., Wang Y., Xing L., He Q., Du H. (2022). Plant pan-genomics: Recent advances, new challenges, and roads ahead. J Genet Genomics..

[bib50] Li X., Huo L., Li X., Zhang C., Gu M., Fan J., Xu C., Gong J., Hu X., Zheng Y., Sun X. (2024). Genomes of diverse *Actinidia* species provide insights into cis-regulatory motifs and genes associated with critical traits. BMC Biol..

[bib51] Li X., Dai X., He H., Lv Y., Yang L., He W., Liu C., Wei H., Liu X., Yuan Q. (2024). A pan-TE map highlights transposable elements underlying domestication and agronomic traits in Asian rice. Natl. Sci. Rev..

[bib52] Liu H., Wang X., Liu S., Huang Y., Guo Y.-X., Xie W.-Z., Liu H., Tahir Ul Qamar M., Xu Q., Chen L.-L. (2022). Citrus Pan-Genome to Breeding Database (CPBD): A comprehensive genome database for citrus breeding. Mol. Plant.

[bib53] Liu Y., Du H., Li P., Shen Y., Peng H., Liu S., Zhou G.-A., Zhang H., Liu Z., Shi M. (2020). Pan-Genome of Wild and Cultivated Soybeans. Cell.

[bib54] Liu Z., Wang N., Su Y., Long Q., Peng Y., Shangguan L., Zhang F., Cao S., Wang X., Ge M. (2024). Grapevine pangenome facilitates trait genetics and genomic breeding. Nat. Genet..

[bib55] Lv Y., Liu C., Li X., Wang Y., He H., He W., Chen W., Yang L., Dai X., Cao X. (2024). A centromere map based on super pan-genome highlights the structure and function of rice centromeres. J. Integr. Plant Biol..

[bib56] Miao H., Wang L., Qu L., Liu H., Sun Y., Le M., Wang Q., Wei S., Zheng Y., Lin W. (2024). Genomic evolution and insights into agronomic trait innovations of *Sesamum* species. Plant Commun..

[bib57] Naish M., Alonge M., Wlodzimierz P., Tock A.J., Abramson B.W., Schmücker A., Mandáková T., Jamge B., Lambing C., Kuo P. (2021). The genetic and epigenetic landscape of the *Arabidopsis* centromeres. Science.

[bib58] Ou L., Li D., Lv J., Chen W., Zhang Z., Li X., Yang B., Zhou S., Yang S., Li W. (2018). Pan-genome of cultivated pepper (*Capsicum*) and its use in gene presence-absence variation analyses. New Phytol..

[bib59] Pinosio S., Giacomello S., Faivre-Rampant P., Taylor G., Jorge V., Le Paslier M.C., Zaina G., Bastien C., Cattonaro F., Marroni F., Morgante M. (2016). Characterization of the poplar pan-genome by genome-wide identification of structural variation. Mol. Biol. Evol..

[bib60] Qiao Q., Edger P.P., Xue L., Qiong L., Lu J., Zhang Y., Cao Q., Yocca A.E., Platts A.E., Knapp S.J. (2021). Evolutionary history and pan-genome dynamics of strawberry (Fragaria spp.). Proc. Natl. Acad. Sci. USA.

[bib61] Qin P., Lu H., Du H., Wang H., Chen W., Chen Z., He Q., Ou S., Zhang H., Li X. (2021). Pan-genome analysis of 33 genetically diverse rice accessions reveals hidden genomic variations. Cell.

[bib62] Rakocevic G., Semenyuk V., Lee W.P., Spencer J., Browning J., Johnson I.J., Arsenijevic V., Nadj J., Ghose K., Suciu M.C. (2019). Fast and accurate genomic analyses using genome graphs. Nat. Genet..

[bib63] Raza A., Bohra A., Garg V., Varshney R.K. (2023). Back to wild relatives for future breeding through super-pangenome. Mol. Plant.

[bib64] Rijzaani H., Bayer P.E., Rouard M., Doležel J., Batley J., Edwards D. (2022). The pangenome of banana highlights differences between genera and genomes. Plant Genome.

[bib65] Satterlee J.W., Alonso D., Gramazio P., Jenike K.M., He J., Arrones A., Villanueva G., Plazas M., Ramakrishnan S., Benoit M. (2024). Convergent evolution of plant prickles by repeated gene co-option over deep time. Science.

[bib66] Schreiber M., Jayakodi M., Stein N., Mascher M. (2024). Plant pangenomes for crop improvement, biodiversity and evolution. Nat. Rev. Genet..

[bib67] Shang L., He W., Wang T., Yang Y., Xu Q., Zhao X., Yang L., Zhang H., Li X., Lv Y. (2023). A complete assembly of the rice Nipponbare reference genome. Mol. Plant.

[bib68] Shang L., Li X., He H., Yuan Q., Song Y., Wei Z., Lin H., Hu M., Zhao F., Zhang C. (2022). A super pan-genomic landscape of rice. Cell Res..

[bib69] Shi J., Tian Z., Lai J., Huang X. (2023). Plant pan-genomics and its applications. Mol. Plant.

[bib70] Shi T., Zhang X., Hou Y., Jia C., Dan X., Zhang Y., Jiang Y., Lai Q., Feng J., Feng J. (2024). The super-pangenome of *Populus unveils* genomic facets for its adaptation and diversification in widespread forest trees. Mol. Plant.

[bib71] Song B., Song Y., Fu Y., Kizito E.B., Kamenya S.N., Kabod P.N., Liu H., Muthemba S., Kariba R., Njuguna J. (2019). Draft genome sequence of *Solanum aethiopicum* provides insights into disease resistance, drought tolerance, and the evolution of the genome. GigaScience.

[bib72] Song J.M., Xie W.Z., Wang S., Guo Y.X., Koo D.H., Kudrna D., Gong C., Huang Y., Feng J.W., Zhang W. (2021). Two gap-free reference genomes and a global view of the centromere architecture in rice. Mol. Plant.

[bib73] Song Y., Han S., Wang M., Ni X., Huang X., Zhang Y. (2024). Pangenome identification and analysis of terpene synthase gene family members in *Gossypium*. Int. J. Mol. Sci..

[bib74] Su Y., Yang X., Wang Y., Li J., Long Q., Cao S., Wang X., Liu Z., Huang S., Chen Z. (2024). Phased telomere-to-telomere reference genome and pangenome reveal an expansion of resistance genes during apple domestication. Plant Physiol..

[bib75] Sun X., Jiao C., Schwaninger H., Chao C.T., Ma Y., Duan N., Khan A., Ban S., Xu K., Cheng L. (2020). Phased diploid genome assemblies and pan-genomes provide insights into the genetic history of apple domestication. Nat. Genet..

[bib76] Sun Y., Shang L., Zhu Q.H., Fan L., Guo L. (2022). Twenty years of plant genome sequencing: Achievements and challenges. Trends Plant Sci..

[bib77] Sun Y., Kou D.-R., Li Y., Ni J.-P., Wang J., Zhang Y.-M., Wang Q.-N., Jiang B., Wang X., Sun Y.-X. (2023). Pan-genome of *Citrullus* genus highlights the extent of presence/absence variation during domestication and selection. BMC Genom..

[bib78] Tao Y., Luo H., Xu J., Cruickshank A., Zhao X., Teng F., Hathorn A., Wu X., Liu Y., Shatte T. (2021). Extensive variation within the pan-genome of cultivated and wild sorghum. Nat. Plants.

[bib79] Tettelin H., Masignani V., Cieslewicz M.J., Donati C., Medini D., Ward N.L., Angiuoli S.V., Crabtree J., Jones A.L., Durkin A.S. (2005). Genome analysis of multiple pathogenic isolates of *Streptococcus agalactiae*: Implications for the microbial "pan-genome. Proc. Natl. Acad. Sci. USA.

[bib80] Varshney R.K., Roorkiwal M., Sun S., Bajaj P., Chitikineni A., Thudi M., Singh N.P., Du X., Upadhyaya H.D., Khan A.W. (2021). A chickpea genetic variation map based on the sequencing of 3,366 genomes. Nature.

[bib81] Wang J., Yang W., Zhang S., Hu H., Yuan Y., Dong J., Chen L., Ma Y., Yang T., Zhou L. (2023). A pangenome analysis pipeline provides insights into functional gene identification in rice. Genome Biol..

[bib82] Wang M., Li J., Qi Z., Long Y., Pei L., Huang X., Grover C.E., Du X., Xia C., Wang P. (2022). Genomic innovation and regulatory rewiring during evolution of the cotton genus *Gossypium*. Nat. Genet..

[bib83] Wang S., Qian Y.Q., Zhao R.P., Chen L.L., Song J.M. (2023). Graph-based pan-genomes: increased opportunities in plant genomics. J. Exp. Bot..

[bib84] Wang T., He W., Li X., Zhang C., He H., Yuan Q., Zhang B., Zhang H., Leng Y., Wei H. (2023). A rice variation map derived from 10 548 rice accessions reveals the importance of rare variants. Nucleic Acids Res..

[bib85] Wei H., Wang X., Zhang Z., Yang L., Zhang Q., Li Y., He H., Chen D., Zhang B., Zheng C. (2024). Uncovering key salt-tolerant regulators through a combined eQTL and GWAS analysis using the super pan-genome in rice. Natl. Sci. Rev..

[bib86] Wei X., Qiu J., Yong K., Fan J., Zhang Q., Hua H., Liu J., Wang Q., Olsen K.M., Han B., Huang X. (2021). A quantitative genomics map of rice provides genetic insights and guides breeding. Nat. Genet..

[bib87] Wei X., Chen M., Zhang Q., Gong J., Liu J., Yong K., Wang Q., Fan J., Chen S., Hua H. (2024). Genomic investigation of 18,421 lines reveals the genetic architecture of rice. Science.

[bib88] Wu D., Xie L., Sun Y., Huang Y., Jia L., Dong C., Shen E., Ye C.-Y., Qian Q., Fan L. (2023). A syntelog-based pan-genome provides insights into rice domestication and de-domestication. Genome Biol..

[bib89] Wu S., Sun H., Gao L., Branham S., McGregor C., Renner S.S., Xu Y., Kousik C., Wechter W.P., Levi A., Fei Z. (2023). A *Citrullus* genus super-pangenome reveals extensive variations in wild and cultivated watermelons and sheds light on watermelon evolution and domestication. Plant Biotechnol. J..

[bib90] Wu Y., Li D., Hu Y., Li H., Ramstein G.P., Zhou S., Zhang X., Bao Z., Zhang Y., Song B. (2023). Phylogenomic discovery of deleterious mutations facilitates hybrid potato breeding. Cell.

[bib91] Yang T., Liu R., Luo Y., Hu S., Wang D., Wang C., Pandey M.K., Ge S., Xu Q., Li N. (2022). Improved pea reference genome and pan-genome highlight genomic features and evolutionary characteristics. Nat. Genet..

[bib92] Zhang B., Zhu W., Diao S., Wu X., Lu J., Ding C., Su X. (2019). The poplar pangenome provides insights into the evolutionary history of the genus. Commun. Biol..

[bib93] Zhang F., Xue H., Dong X., Li M., Zheng X., Li Z., Xu J., Wang W., Wei C. (2022). Long-read sequencing of 111 rice genomes reveals significantly larger pan-genomes. Genome Res..

[bib94] Zhang F., Xue H., Dong X., Li M., Zheng X., Li Z., Xu J., Wang W., Wei C. (2022). Long-read sequencing of 111 rice genomes reveals significantly larger pan-genomes. Genome Res..

[bib95] Zhang H., Chen W., Zhu D., Zhang B., Xu Q., Shi C., He H., Dai X., Li Y., He W. (2024). Population-level exploration of alternative splicing and its unique role in controlling agronomic traits of rice. Plant Cell.

[bib96] Zhang Y., Zhao M., Tan J., Huang M., Chu X., Li Y., Han X., Fang T., Tian Y., Jarret R. (2024). Telomere-to-telomere *Citrullus* super-pangenome provides direction for watermelon breeding. Nat. Genet..

[bib97] Zhao Q., Feng Q., Lu H., Li Y., Wang A., Tian Q., Zhan Q., Lu Y., Zhang L., Huang T. (2018). Pan-genome analysis highlights the extent of genomic variation in cultivated and wild rice. Nat. Genet..

[bib98] Zhao Q., Feng Q., Lu H., Li Y., Wang A., Tian Q., Zhan Q., Lu Y., Zhang L., Huang T. (2018). Pan-genome analysis highlights the extent of genomic variation in cultivated and wild rice. Nat. Genet..

[bib99] Zhou Y., Zhang Z., Bao Z., Li H., Lyu Y., Zan Y., Wu Y., Cheng L., Fang Y., Wu K. (2022). Graph pangenome captures missing heritability and empowers tomato breeding. Nature.

[bib100] Zhou Y., Yu Z., Chebotarov D., Chougule K., Lu Z., Rivera L.F., Kathiresan N., Al-Bader N., Mohammed N., Alsantely A. (2023). Pan-genome inversion index reveals evolutionary insights into the subpopulation structure of Asian rice. Nat. Commun..

[bib101] Zhuang Y., Wang X., Li X., Hu J., Fan L., Landis J.B., Cannon S.B., Grimwood J., Schmutz J., Jackson S.A. (2022). Phylogenomics of the genus Glycine sheds light on polyploid evolution and life-strategy transition. Nat. Plants.

